# High stereoselectivity on low temperature Diels-Alder reactions

**DOI:** 10.1186/1860-5397-1-14

**Published:** 2005-12-09

**Authors:** Luiz Carlos da Silva Filho, Valdemar Lacerda Júnior, Mauricio Gomes Constantino, Gil Valdo José da Silva, Paulo Roberto Invernize

**Affiliations:** 1 Departamento de Química, Faculdade de Filosofia, Ciências e Letras de Ribeirão Preto, Universidade de São Paulo, Av. Bandeirantes 3900, 14040-901 - Ribeirão Preto - SP, Brazil

## Abstract

We have found that some of the usually poor dienophiles (2-cycloenones) can undergo Diels-Alder reaction at -78°C with unusually high stereoselectivity in the presence of niobium pentachloride as a Lewis acid catalyst. A remarkable difference in reaction rates for unsubstituted and α- or β-methyl substituted 2-cycloenones was also observed.

## Introduction

For more than 70 years, the Diels-Alder reaction, or [4+2] cycloaddition reaction, has remained as one of the best powerful organic transformations in chemical synthesis, particularly in obtaining polycyclic rings. Many factors have contributed to the popularity of this reaction in organic synthesis (e.g., rapid accumulation of polyfunctionality and stereochemical control). In most cases the cycloaddition proceeds quite well by simply mixing the substrates, because the usual dienophiles have a carbonyl or equivalent group that, through conjugation, lower the energy of the LUMO antibonding π orbital to an appropriate level for reaction with the diene HOMO. In some cases, however, as it happens with cycloenones, this energy lowering is not enough to produce a reaction, and it was early realized that Lewis acids, usually AlCl_3_, could be used to enhance the reactivity of these poor dienophiles. [1–4] Particularly the cycloenones are desirable dienophiles, because their cycloaddition reactions are a remarkable tool for the synthesis of octalones and related bicyclic olefinic ketones (e.g., decaline-based sesquiterpenes, labdanic and hydrophenanthroid diterpenes, steroids, and tetracyclic and pentacyclic triterpenes) [5–6].

The role of the Lewis acid is to produce an extra lowering of the LUMO energy of the carbonyl substrate, through complexation with the carbonyl oxygen thus reducing the electron density of the double bond. Moreover, the coordination of the Lewis acid to the carbonyl oxygen increases the magnitude of the coefficients at the carbonyl and at the β-carbon in the π* C = C-C = O orbital, increasing secondary orbital interactions and rendering the molecule more susceptible to nucleophilic attack. The resulting lowering of the activation energy usually leads to an enhancement of stereo- and regioselectivity [7].

It was also soon realized that the modifications in the cycloaddition reactions parameters should depend on the Lewis acids used, and a number of authors have performed comparison studies [8–11] that demonstrated how large the influence of the Lewis acid can have on several aspects of the reactions.

As part of our research work on synthetic methodologies using niobium pentachloride in a variety of reactions [12–14], we have recently started an investigation on catalyzed Diels-Alder reactions [15]. Cycloenones usually do not react with cyclopentadiene in absence of a Lewis acid. In our previous exploratory experiments we have demonstrated that niobium pentachloride promotes Diels-Alder reaction, in ethyl acetate solution, between 2-cyclohexenone (**2**) and cyclopentadiene with high stereoselectivity at -78°C (only the *endo* adduct was obtained). Motivated by this result we decided to investigate the reactions of various 2-cycloenones **1** – **6** (dienophiles) with cyclopentadiene, in the presence of NbCl_5_, at different temperatures. Rather surprisingly, however, we found that ethyl acetate as solvent, efficient for **2**, did not work with cycloenone **3**. By using ethyl ether we obtained good results in both cases, so this work was all performed using ethyl ether as solvent.

## Results and discussion

Reactions were performed in ethyl ether solution, at 3 different temperatures: -78°C, room temperature, or under reflux. Excess of diene (cyclopentadiene) was used in each experiment (5 eq.), while the molar ratio dienophile/NbCl_5_ was maintained constant at 1.0/0.5. The results obtained in these studies are summarized in Table 1

**Table 1:** Diels-Alder reactions of 2-cycloenones **1 – 6** with cyclopentadiene catalyzed by NbCl_5_.

**Cycloenone**	**Products**	**Conversion (%)**	**Temp. (°C)**	**Time**	**Yield,% ^a^**	**Ratio^b^**	
						
						***endo***	***exo***

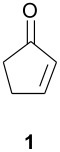	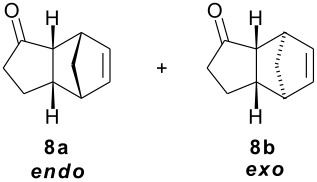	100 100 100	-78 rt reflux	3 h 25 min 5 min	61 58 65	89 78 74	11 22 24

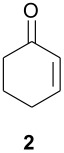	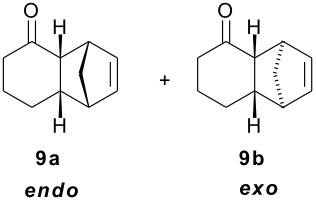	100 100 100	-78 rt reflux	3 h 45 min 15 min	72 58 62	100 80 78	0 20 22

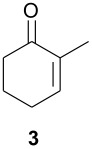	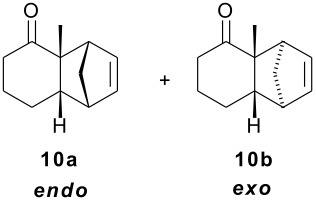	6 45 100	-78 rt reflux	8 h 24 h 12 h	32 43 65	48 42 30	52 58 70

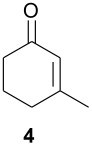	NO REACTION	0 0 0	-78 rt reflux	8 h 24 h 24 h	-- -- --	-- -- --	

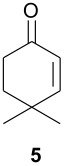	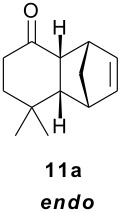	22 50 100	-78 rt reflux	8 h 24 h 24 h	40 34 48	100 100 100	0 0 0

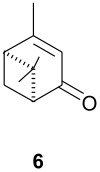	NO REACTION	0 0 0	-78 rt reflux	8 h 24 h 24 h	-- -- --	-- -- --	

^a^ Isolated yield. In cases where the starting material was partially recovered, the yield was calculated based on the amount of starting material effectively transformed. ^b^ *endo* – *exo* ratios determined by ^1^H NMR of the crude reaction product.

All products were isolated and characterized by spectroscopic and spectrometric methods (^1^H NMR, ^13^C NMR, IR and mass spectra). A detailed NMR analysis and theoretical calculations with total assignment of ^1^H and ^13^C NMR data for compounds **9a** and **9b** was recently published [16].

As observed in Table 1, unsubstituted 2-cycloenones **1** and **2** react well with cyclopentadiene, giving the corresponding *endo* and *exo* adducts in a ratio depending on the reaction temperature; as expected, lower temperature result in higher selectivity. Methyl substitution in cycloenones has a remarkable influence on reactivity: α-methylated (**3**) and γ,γ-dimethylated (**5**) 2-cycloenones are far less reactive, while β-methylated cycloenones (**4** and **6**) did not react at all.

The rather perplexing low reactivity of β-methylated cycloenones was already observed by Wenkert et al. [17]. We are presently carrying out HOMO-LUMO and transition state calculations, seeking for the reasons that could explain these results.

A remarkable aspect of this work is the higher efficiency of NbCl_5_, as compared to other Lewis acid catalysts. Lower required times and temperatures, lower diene excess and higher stereoselectivity were observed in most cases. Table 2 shows some examples comparing our results for 2-cycloenones **2**, **3** and **5** with literature data using other Lewis acids.

**Table 2:** Comparison between Diels-Alder reactions of 2-cycloenones with cyclopentadiene catalyzed by NbCl_5_, AlCl_3_ [1] and SnCl_4_ [18].

a) For Cycloenone **2**								

**Lewis acid**	**Diene (equivalents)**	**Lewis acid (equivalents)**	**Solvent**	**Time**	**Temp. (°C)**	**Yield, %**	**Ratio**	
							
							***endo***	***exo***

NbCl_5_	5	0.5	Et_2_O	3 h	-78	72	100	0
				45 min.	rt	58	80	20
				15 min.	reflux	62	78	22

AlCl_3_ [1]	6	0.25	Toluene	7 h	40	80	89	11

SnCl_4_ [18]	50	1.0	CH_2_Cl_2_	14 h	-20	93	92	8

b) For Cycloenone **3**								

**Lewis acid**	**Diene (equivalents)**	**Lewis acid (equivalents)**	**Solvent**	**Time**	**Temp. (°C)**	**Yield, %**	**Ratio**	
							
							***endo***	***exo***

NbCl_5_	5	0.5	Et_2_O	8 h	-78	32	48	52
				24 h	rt	43	42	58
				12 h	reflux	65	30	70

AlCl_3_ [1]	15	0.25	Toluene	20 h	40	70	30	70

c) For Cycloenone **5**								

**Lewis acid**	**Diene (equivalents)**	**Lewis acid (equivalents)**	**Solvent**	**Time**	**Temp. (°C)**	**Yield, %**	**Ratio**	
							
							***endo***	***exo***

NbCl_5_	5	0.5	Et_2_O	8 h	-78	40	100	0
				24 h	rt	34	100	0
				5 h	reflux	48	100	0

AlCl_3_ [1]	6	0.25	Toluene	20 h	40	92	95	5

The possibility of effecting Diels-Alder reactions at -78°C with some substrates is, in our opinion, the most important aspect: besides demonstrating the strong activation of the 2-cycloenones system exerted by NbCl_5_, it results in the expected higher selectivity. However, for 2-cycloenone **3**, we observe an apparently higher selectivity at higher temperature. This can be due to kinetic/thermodynamic competition, as is observed for many reactions.

The unusually high *endo*/*exo* selectivity for 2-cycloenone **5** has been previously observed in reactions with AlCl_3_ [1,3]. Comparing NbCl_5_ results for **5** and **2** (Table 1), we can observe that the methyl groups of **5** produce an enhancement of selectivity, possibly through some kind of interaction with the methylene group of cyclopentadiene destabilizing the transition state corresponding to the *exo* product. This would increase the usual difference between the two transition states, normally due mainly to π orbital overlaps. As already mentioned, we are performing HOMO-LUMO and transition states calculations to verify some structural factors influences on the course of these reactions.

## Conclusion

NbCl_5_ has proved to be a promising tool for Diels-Alder reactions between 2-cycloenones and cyclopentadiene. As compared to other Lewis acids, it is more effective, giving higher stereoselectivity, good yields and requiring lower reaction times and temperatures.

## Experimental section

### Preparation of substrates

#### 2-Methyl-2-cyclohexen-1-one (3) [19]

To a solution of 2-methyl-cyclohexanone (3.4 g, 30.3 mmol) in 40.0 mL of CCl_4_ was added 5.3 g of (NBS) N-bromo-succicinimide (30.0 mmol). The mixture was stirred and refluxed for 4 h heating with a 200 W tungsten lamp. The reaction was cooled, filtered and the solvent was removed under vacuum. The resulting oil was dissolved in 10.0 mL of anhydrous pyridine and refluxed for 12 h. The mixture was cooled, diluted with water and extracted with ethyl ether (3 × 5.0 mL). The organic layer was washed with a 10% aqueous solution of CuSO_4_ (3 × 5.0 mL), dried over anhydrous magnesium sulfate and the solvent was removed under vacuum. The product was distilled in a *short-path* at 80°C (40 mmHg). Yield of compound **3** as colorless oil: 1.20 g (36%):

#### 3-Methyl-2-cyclohexen-1-one (4) [20]

Prepared as described in reference, using 12.6 g of ethyl acetoacetate. Yield of compound **4** as pale yellow oil: 6.82 g (64%):

#### 4,4-Dimethyl-2-cyclohexen-1-one (5) [21]

Prepared as described in reference, using 12.3 g of isobutyraldehyde. Yield of compound **5** as a colorless liquid 8.47 g (68%), b.p. 73–74° (14 mmHg).

### General procedure for the reactions of cycloenones and cyclopentadiene with NbCl_5_

To a solution of niobium pentachloride (0.135 g, 0.5 mmol) in 1.0 mL of anhydrous ethyl ether, maintained at room temperature, reflux or -78°C under nitrogen atmosphere, was added a solution of the cycloenone (1.0 mmol) and cyclopentadiene (5 mmols) in 1.0 mL of anhydrous ethyl ether. The reaction mixture was quenched with a 10% aqueous citric acid solution (2.0 mL, when working at room temperature or reflux) or with a 1:1 solution of water/THF (2.0 mL, when working at -78°C). The mixture was diluted with water (5.0 mL) and solvent (10.0 mL), the organic layer was separated and washed with 5% aqueous sodium bicarbonate (3 × 10.0 mL), saturated brine (2 × 10.0 mL), and dried over anhydrous magnesium sulfate. The solvent was removed under vacuum and the products were purified by column chromatography through silica gel using mainly a mixture of hexane and ethyl acetate (9.5:0.5) as eluent.

## Supporting Information

File 1 Contains general methods (S2), characterization data for compounds 3, 4, 8ab, 9ab, 10ab, and 11a (S3-S6).

File 2 ^1^H and ^13^C NMR spectra of compounds 3, 4, 8ab, 9ab, 10ab, and 11a. (S2-S19).
