# Supplementary material for: High stereoselectivity on low temperature Diels-Alder reactions
Source: Beilstein J Org Chem. 2005 Dec 9;1:14. doi: 10.1186/1860-5397-1-14 (PMC1399462; doi:10.1186/1860-5397-1-14)
Supplement: File 1 — Contains general methods (S2), characterization data for compounds 3, 4, 8ab, 9ab, 10ab, and 11a (S3-S6). [file Beilstein_J_Org_Chem-01-14-s001.doc]

**Supplementary Material**

**High Stereoselectivity on Low Temperature Diels-Alder Reactions**

Luiz Carlos da Silva Filho, Valdemar Lacerda Júnior,* Mauricio Gomes Constantino, Gil Valdo José da Silva and Paulo Roberto Invernize

Departamento de Química, Faculdade de Filosofia, Ciências e Letras de Ribeirão Preto, Universidade de São Paulo, Av. Bandeirantes 3900, 14040-901 – Ribeirão Preto – SP, Brazil.

E-mail: Luiz Carlos da Silva Filho – [lcsilva@aluno.ffclrp.usp.br](mailto:lcsilva@aluno.ffclrp.usp.br); Valdemar Lacerda Júnior – [vljunior@usp.br](mailto:vljunior@usp.br); Mauricio Gomes Constantino - [mgconsta@usp.br](mailto:mgconsta@usp.br); Gil Valdo José da Silva – [gvjdsilv@usp.br](mailto:gvjdsilv@usp.br); Paulo Roberto Invernize – [paulinver@bol.com.br](mailto:paulinver@bol.com.br)

Table of Contents: Contains general methods (S2), characterization data for compounds 3, 4, 8ab, 9ab, 10ab, and 11a (S3-S6).

**General Methods.** NMR spectra were obtained on a Bruker AVANCE DRX 400 MHz spectrometer equipped with a 5.0 mm direct dual probe (^13^C-^1^H) operating at 400.13 MHz (^1^H) and 100.61 MHz (^13^C) at a temperature of 300 K; deuterochloroform was used as solvent and tetramethylsilane as internal standard. IR spectra were recorded with a Perkin Elmer RX IFTIR System, using KBr pellets, and the most intense or representative bands are reported (in cm^-1^). Mass spectra were determined at an ionizing voltage of 70 eV, using a HP 5988-A spectrometer, with fused silica capillary GC columns (25 m length x 0.2 mm i.d. x 0.33 μm phase thickness). The column temperature was 100 ^o^C (3 min) ramped to 220 ^o^C at 20 ^o^C min^-1^. Analytical gas chromatography (GLC) separations were performed with a fused silica capillary column (30 m length x 0.25 mm i.d.) operating at temperatures in the range 50-200ºC. Column chromatography separations were performed with silica gel (60-230 mesh). Except where specified, all reagents were purchased from commercial sources and were used without further purification.

**2-Methyl-2-cyclohexen-1-one (3)**.^19^ **^1^H NMR** (400 MHz, CDCl_3_): δ 6.75 (tq, 1H, *J*_1_ = 4.2 and *J*_2_ = 1.5 Hz); 2.43 (dd, 2H, *J*_1_ = 6.7 and *J*_2_ = 6.5 Hz); 2.33 (m, 2H); 1,99 (tt, *J*_1_ = 6.7 and *J*_2_ = 6.0 Hz); 1.77 (dd, 3H, *J*_1_ = 3.5 and *J*_2_ = 1.5 Hz); **^13^C NMR** (100 MHz, CDCl_3_): δ 200.1 (C=O); 145.6 (CH); 135.7 (C); 38.3 (CH_2_); 26.0 (CH_2_); 23.3 (CH_2_); 16.0 (CH_3_); **IR** (film) ν_max_: 2923; 2868; 1675; 1431; 1360; 1174; 1107; 1022 cm^-1^; **MS** m/z (rel. intensity) (%): 110(M)^+^ (41); 52 (100); 67 (16); 54 (55); 39 (38); 28 (17).

**3-Methyl-2-cyclohexen-1-one (4).**^20^ **^1^H NMR** (400 MHz, CDCl_3)_: δ 5.86 (s, 1H); 2.32 (m, 4H); 2.01 (m, 2H); 1.97 (s, 3H); **^13^C NMR** (100 MHz, CDCl_3_): δ 200.0 (C=O); 163.2 (C); 126.9 (CH); 37.9 (CH_2_); 31.2 (CH_2_); 24.7 (CH_3_); 22.8 (CH_2_); **IR** (film) ν_max_: 3039; 2963; 2872; 1677; 1629; 1429; 1383; 1255; 1196; 1022 cm^-1^; **MS** m/z (rel. intensity) (%): 110 (M)^+^ (5); 82 (89); 67 (8); 54 (32); 41 (10); 39 (40); 32 (25); 28 (100).

**4,4-Dimethyl-2-cyclohexen-1-one (5)**.^21^ **^1^H NMR** (400 MHz, CDCl_3_): δ 6.66 (dt, 1H, *J*_1_ = 10.1 and *J*_2_ = 0.9 Hz); 5.84 (d, 1H, *J*_1_ = 10.1 Hz); 1.87 (m, 2H); 2.46 (m, 2H); 1.17 (s, 6H); **^13^C NMR** (100 MHz, CDCl_3_): 200.1 (C=O); 160.3 (CH); 127.2 (CH); 36.4 (CH_2_); 34.7 (CH_2_); 33.2 (C); 28.0 (2 CH_3_); **IR** (film) ν_max_: 2960; 2868; 1696; 1681; 1468 cm^-1^; **MS** m/z (rel. intensity) (%):124 (M)^+^; 96 (56); 82 (100); 81 (56); 68 (25); 67 (42); 53 (22); 43 (21); 41 (25); 39 (25).

**(1R,2R,6*R*,7*S*)-Tricyclo[5.2.1.0^2,6^]dec-8-en-3-one (8a)** (pale yellow oil):^22^ **^1^H NMR** (400 MHz, CDCl_3_): δ 6.15 (dd, 1H, *J*_1_ = 5.7 and *J*_2_ = 3.0 Hz); 6.04 (dd, 1H, *J*_1_ = 5.7 and *J*_2_ = 3.0 Hz); 3.13 (br. s, 1H); 2.94 (br. s, 1H); 2.90 (ddd, 1H, *J*_1_ = 9.1; *J*_2_ = 4.3 and *J*_3_ = 3.0 Hz); 2.79 (ddd, 1H, *J*_1_ = 9.1; *J*_2_ = 4.8 and *J*_3_ = 1.7 Hz); 2.07 (m, 1H); 1.94 (ddd, 1H, *J*_1_ = 14.4; *J*_2_ = 9.3 and *J*_3_ = 7.1 Hz); 1.92 (m, 1H); 1.46 (dt, 1H, *J*_1_ = 8.3 and *J*_2_ = 1.5 Hz); 1.45 (m, 1H); 1.35 (dt, 1H, *J*_1_ = 8.3 and *J*_2_ = 1.5 Hz); **^13^C NMR** (100 MHz, CDCl_3_): δ 222.4 (C=O); 136.1 (CH); 134.7 (CH); 54.5 (CH); 52.6 (CH_2_); 47.4 (CH); 47.0 (CH); 41.1 (CH); 40.5 (CH_2_); 22.6 (CH_2_); **IR** (film) ν_max_: 3060; 1740; 1460; 1340; 1231; 1170; 908 cm^-1^.

**(1S,2R,6R,7R)-Tricyclo[5.2.1.0^2,6^]dec-8-en-3-one (8b)** (pale yellow oil):^22^ **^1^H NMR** (400 MHz, CDCl_3_): δ 6.12 (dd, 1H, *J*_1_ = 5.6 and *J*_2_ = 3.0 Hz); 6.09 (dd, 1H, *J*_1_ = 5.6 and *J*_2_ = 3.0 Hz); 3.02 (br. s, 1H); 2.69 (br. s, 1H); 2.48 (dddd, 1H, *J*_1_ = 18.9; *J*_2_ = 11.1; *J*_3_ = 9.2 and *J*_4_ = 1.5 Hz); 2.39–2.44 (m, 2H); 2.19 (dddd, 1H, *J*_1_ = 13.9; *J*_2_ = 11.1; *J*_3_ = 9.2 and *J*_4_ = 5.0 Hz); 2.15 (dd, 1H, *J*_1_ = 8.6 and *J*_2_ = 2.8 Hz); 1.44 (ddd, 1H, *J*_1_ = 18.9; *J*_2_ = 9.2 and *J*_3_ = 5.0 Hz); 1.35 (dt, 1H, *J*_1_ = 9.1 and *J*_2_ = 1.8 Hz); 1.21 (dt, 1H, *J*_1_ = 9.1 and *J*_2_ = 1.6 Hz); **^13^C NMR** (100 MHz, CDCl_3_): δ 222.2 (C=O); 138.3 (CH); 137.2 (CH); 54.0 (CH); 48.8 (CH_2_); 45.8 (CH); 43.6 (CH); 42.4 (CH); 42.0 (CH_2_); 24.6 (CH_2_); **IR** (film) ν_max_: 3060; 1740; 1460; 1340; 1231; 1170; 908 cm^-1^.

**(1R,2R,7R,8S)-Tricyclo[6.2.1.0^2,7^]undec-9-en-3-one (9a)** (colorless oil):^1,16^ **^1^H NMR** (400 MHz, CDCl_3_): δ 6.18 (ddddd, 1H, *J*_1_ = 5.7; *J*_2_ = 2.9; *J*_3_ = 1.0; *J*_4_ = 0.9 and *J*_5_ = 0.4 Hz); 6.01 (ddddd, 1H, *J*_1_ = 5.7; *J*_2_ = 2.9; *J*_3_ = 1.0; *J*_4_ = 0.9 and *J*_5_ = 0.4 Hz); 3.26 (dddddd, 1H, *J*_1_ = 3.5; *J*_2_ = 2.9; *J*_3_ = 2.4; *J*_4_ = 1.7; *J*_5_ = 1.3 and *J*_6_ = 1.0 Hz); 2.88 (dddddd, 1H, *J*_1_ = 3.3; *J*_2_ = 2.9; *J*_3_ = 2.4; *J*_4_ = 1.7; *J*_5_ = 1.3 and *J*_6_ = 1.0 Hz); 2.73 (ddd, 1H, *J*_1_ = 10.4; *J*_2_ = 3.5 and *J*_3_ = 0.6 Hz); 2.67 (dddd, 1H, *J*_1_ = 10.8; *J*_2_ = 10.4; *J*_3_ = 6.3 and *J*_4_ = 3.3 Hz); 2.32 (ddddd, 1H, *J*_1_= 18.6; *J*_2_ = 6.2; *J*_3_ = 2.6; *J*_4_ = 1.7 and *J*_5_ = 0.6 Hz); 1.97 (ddddd, 1H, *J*_1_ = 12.9; *J*_2_ = 6.3; *J*_3_ = 4.7; *J*_4_ = 2.9 and *J*_5_ = 1.7 Hz); 1.93 (ddd, 1H, *J*_1_ = 18.6; *J*_2_ = 11.6 and *J*_3_ = 7.1 Hz); 1.79 (ddddd, 1H, *J*_1_ = 13.7; *J*_2_ = 7.1; *J*_3_ = 4.7; *J*_4_ = 3.1 and *J*_5_ = 2.6 Hz); 1.70 (ddddd, 1H, *J*_1_ =13.7; *J*_2_ = 12.9; *J*_3_ =11.6; *J*_4_ = 6.2 and *J*_5_ = 2.9 Hz); 1.45 (dtt, 1H, *J*_1_ = 8.3; *J*_2_ = 1.7 and *J*_3_ = 0.4 Hz); 1.31 (dtt, 1H, *J*_1_ = 8.3; *J*_2_ = 2.4 and *J*_3_ = 0.9 Hz); 0.77 (ddt, 1H, *J*_1_ = 12.9; *J*_2_ = 10.8 and *J*_3_ = 3.1 Hz); **^13^C NMR** (100 MHz, CDCl_3_): δ 215.5 (C=O); 137.7 (CH); 135.0 (CH); 51.7 (CH); 48.4 (CH_2_); 46.6 (CH); 45.2 (CH); 41.4 (CH); 39.4 (CH_2_); 28.1 (CH_2_); 21.8 (CH_2_); **IR** (film) ν_max_: 2935; 2866; 1734; 1699; 1336; 1236; 1173; 910; 733 cm^-1^; **MS** m/z (rel. intensity) (%):162 (M)^+^ (2); 121 (2); 97 (39); 91 (19); 79 (15); 66 (100); 43 (8); 41 (16).

**(1S,2R,7R,8R)-Tricyclo[6.2.1.0^2,7^]undec-9-en-3-one (9b)** (colorless oil):^1,16^ **^1^H NMR** (400 MHz, CDCl_3_): δ 6.11 (ddt, 1H, *J*_1_ = 5.7; *J*_2_ = 3.1 and *J*_3_ = 1.0 Hz); 6.03 (ddt, 1H, *J*_1_ = 5.7; *J*_2_ = 3.1 and *J*_3_ = 1.0 Hz); 3.25 (ddddd, 1H, *J*_1_ = 3.1; *J*_2_ = 1.8; *J*_3_ = 1.8; *J*_4_ = 1.3 and *J*_5_ = 1.0 Hz); 2.61 (ddddd, 1H, *J*_1_ = 3.1; *J*_2_ = 1.8; *J*_3_ = 1.8; *J*_4_ = 1.3 and *J*_5_ = 1.0 Hz); 2.46 (dddt, 1H, *J*_1_ = 18.4; *J*_2_ = 7.6; *J*_3_ = 1.8 and *J*_4_ = 0.5 Hz); 2.22 (ddd, 1H, *J*_1_ = 18.4; *J*_2_ = 10.9 and *J*_3_ = 7.9 Hz); 2.01 (ddddd, 1H, *J*_1_ = 13.7; *J*_2_ = 6.1; *J*_3_ = 4.6; *J*_4_ = 3.8 and *J*_5_ = 2.6 Hz); 1,96 (dddd, 1H, *J*_1_ = 9.3; *J*_2_ = 3.8; *J*_3_ = 1.8 and *J*_4_ = 0.5 Hz); 1,94 (dddd, 1H, *J*_1_ = 11.2; *J*_2_ = 9.3; *J*_3_ = 6.1 and *J*_4_ = 1.8 Hz); 1.87 (ddddd, 1H, *J*_1_ = 13.7; *J*_2_ = 7.9; *J*_3_ = 4.6; *J*_4_ = 2.8 and *J*_5_ = 1.8 Hz); 1.69 (ddddd, 1H, *J*_1_ = 13.7; *J*_2_ = 13.4; *J*_3_ = 10.9; *J*_4_ = 7.6 and *J*_5_ = 2.6 Hz); 1.23 (dq, 1H, *J*_1_ = 9.0 and *J*_2_ = 1.8 Hz); 1.11 (dtt, 1H, *J*_1_ = 9.0; *J*_2_ = 1.5 and *J*_3_ = 1.0 Hz); 0.87 (dddd, 1H, *J*_1_ = 13.7; *J*_2_ = 13.4; *J*_3_ = 11.2 and *J*_4_ = 2.8 Hz); **^13^C NMR** (100 MHz, CDCl_3_): δ 215.5 (C=O); 137.8 (CH); 135.5 (CH); 50.3 (CH); 47.0 (CH_2_); 44.5 (CH); 44.2 (CH); 41.4 (CH); 39.2 (CH_2_); 29.7 (CH_2_); 21.7 (CH_2_); **IR** (film) ν_max_: 2930; 2864; 1734; 1695; 1336; 1235; 1173; 908; 730 cm^-1^; **MS** m/z (rel. intensity) (%): 162 (M)^+^ (2); 121 (3); 97 (52); 91 (15); 79 (11); 66 (100); 51 (7).

**(1R,2R,7R,8S)-2-Metil-tricyclo[6.2.1.0^2,7^]undec-9-en-3-one (10a)** (pale yellow oil):^1^ **^1^H NMR** (400 MHz, CDCl_3_): δ 6.18 (dd, 1H, *J*_1_ = 5.8 and *J*_2_ = 3.0 Hz); 5.99 (dd, 1H, *J*_1_ = 5.8 and *J*_2_ = 3.0 Hz); 2.85 (br. s, 1H); 2.77 (br. s, 1H); 2.40 (ddt, 1H, *J*_1_ = 18.9; *J*_2_ = 5.8 and *J*_3_ = 2.3 Hz); 2.13 (ddd, 1H, *J*_1_ = 10.9; *J*_2_ = 6.8 and *J*_3_ = 3.3 Hz); 1.99 (m, 1H); 1.92 (ddd, 1H, *J*_1_ = 18.9; *J*_2_ = 11.6 and *J*_3_ = 6.8 Hz); 1.68-1.83 (m, 3H); 1.55 (d, 1H, *J* = 8.6 Hz); 1.43 (dt, 1H, *J*_1_ = 8.6 and *J*_2_ = 1.8 Hz); 1.29 (s, 3H).

**(1S,2R,7R,8R)-2-Metil-tricyclo[6.2.1.0^2,7^]undec-9-en-3-one (10b)** (pale yellow oil):^1^ **^1^H NMR** (400 MHz, CDCl_3_): δ 6.28 (dd, 1H, *J*_1_ = 5.8 and *J*_2_ = 3.0 Hz); 6.07 (dd, 1H, *J*_1_ = 5.8 and *J*_2_ = 3.0 Hz); 3.19 (br. s, 1H); 2.61 (ddt, 1H, *J*_1_ = 18.9; *J*_2_ = 7.1 and *J*_3_ = 1.9 Hz); 2.48 (br. s, 1H); 2.23 (ddd, 1H, *J*_1_ = 18.9; *J*_2_ = 11.3 and *J*_3_ =7.7 Hz); 2.07 (m, 1H); 1.91 (m, 1H); 1.79 (m, 2H); 1.48 (ddd, 1H, *J*_1_ = 11.9; *J*_2_ = 6.6 and *J*_3_ = 1.8 Hz); 1.35 (dt, 1H, *J*_1_ = 9.1 and *J*_2_ = 1.8 Hz); 1.24 (d, 1H, *J* = 9.1 Hz); 1.01 (s, 3H).

**(1R,2S,7R,8S)-6,6-Dimethyl-tricyclo[6.2.1.0^2,7^]undec-8-en-3-one (11a)** (colorless oil):^1^ **^1^H NMR** (400 MHz, CDCl_3_): δ 6.07 (dd, 1H, *J*_1_ = 5.6 and *J*_2_ = 2.8 Hz); 6.02 (dd, 1H, *J*_1_ = 5.6 and *J*_2_ = 2.8 Hz); 3.16 ( br. s, 1H); 2.91 (br. s, 1H); 2.76 (ddt, 1H, *J*_1_ = 9.6; *J*_2_ = 4.3; *J*_3_ = 1.0 Hz); 2.23 (ddd, 1H, *J*_1_ = 9.6; *J*_2_ = 3.1 and *J*_3_ = 1.4 Hz); 2.14 (dddd, 1H, *J*_1_ = 18.9; *J*_2_ = 10.1; *J*_3_ = 7.5 and *J*_4_ = 1.4 Hz); 1.97 (dddd, 1H, *J*_1_ = 18.9; *J*_2_ = 6.4; *J*_3_ = 4.3 and *J*_4_ = 0.9 Hz); 1.50 (ddd, 1H, *J*_1_ = 13.6; *J*_2_ = 10.1 and *J*_3_ = 6.4 Hz); 1.36 (dt, 1H, *J*_1_ = 8.3 and *J*_2_ = 1.8 Hz); 1.27 (dddd, 1H, *J*_1_ = 13.6; *J*_2_ = 7.5; *J*_3_ = 4.3 and *J*_4_ = 1.5 Hz); 1.23 (dt, 1H, *J*_1_ = 8.3; *J*_2_= 1.3 Hz); 0.97 (s, 3H); 0.87 (s, 3H); **^13^C NMR** (100 MHz, CDCl_3_): δ 215.8 (C=O); 136.0 (CH); 135.6 (CH); 51.6 (CH); 51.3 (CH_2_); 50.9 (CH); 47.7 (CH); 46.5 (CH); 36.3 (CH_2_); 33.1 (CH_2_); 31.9 (C); 31.3 (CH_3_); 28.5 (CH_3_); **IR** (film) ν_max_: 2958; 2889; 1738; 1733; 1700; 1456; 1365; 1183; 733 cm^-1^.
