# Supplementary material for: High stereoselectivity on low temperature Diels-Alder reactions
Source: Beilstein J Org Chem. 2005 Dec 9;1:14. doi: 10.1186/1860-5397-1-14 (PMC1399462; doi:10.1186/1860-5397-1-14)

**Supplementary Material**

**High Stereoselectivity on Low Temperature Diels-Alder Reactions**

Luiz Carlos da Silva Filho, Valdemar Lacerda Júnior,* Mauricio Gomes Constantino, Gil Valdo José da Silva and Paulo Roberto Invernize

Departamento de Química, Faculdade de Filosofia, Ciências e Letras de Ribeirão Preto, Universidade de São Paulo, Av. Bandeirantes 3900, 14040-901 – Ribeirão Preto – SP, Brazil.

E-mail: Luiz Carlos da Silva Filho – [lcsilva@aluno.ffclrp.usp.br](mailto:lcsilva@aluno.ffclrp.usp.br); Valdemar Lacerda Júnior – [vljunior@usp.br](mailto:vljunior@usp.br); Mauricio Gomes Constantino - [mgconsta@usp.br](mailto:mgconsta@usp.br); Gil Valdo José da Silva – [gvjdsilv@usp.br](mailto:gvjdsilv@usp.br); Paulo Roberto Invernize – [paulinver@bol.com.br](mailto:paulinver@bol.com.br)

Table of Contents: ^1^H and ^13^C NMR spectra of compounds 3, 4, 8ab, 9ab, 10ab, and 11a. (S2-S19).


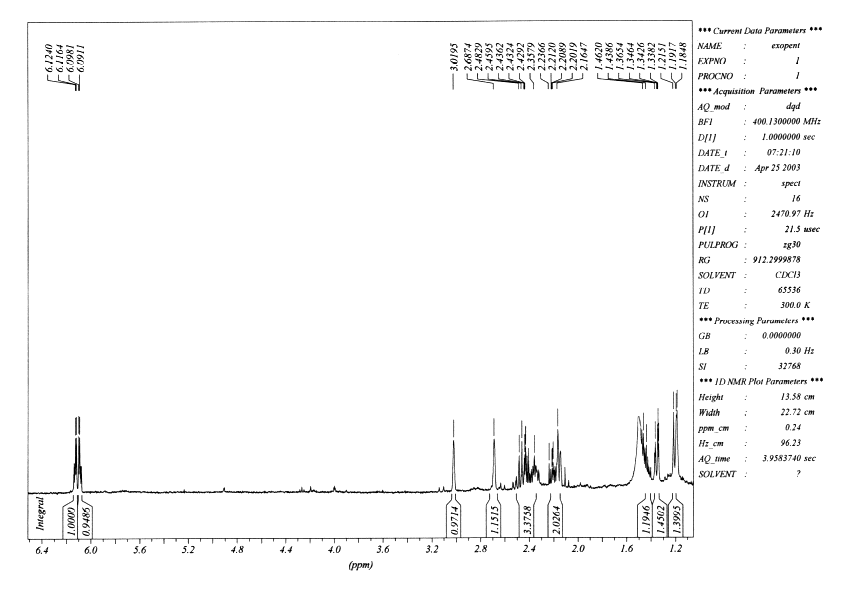


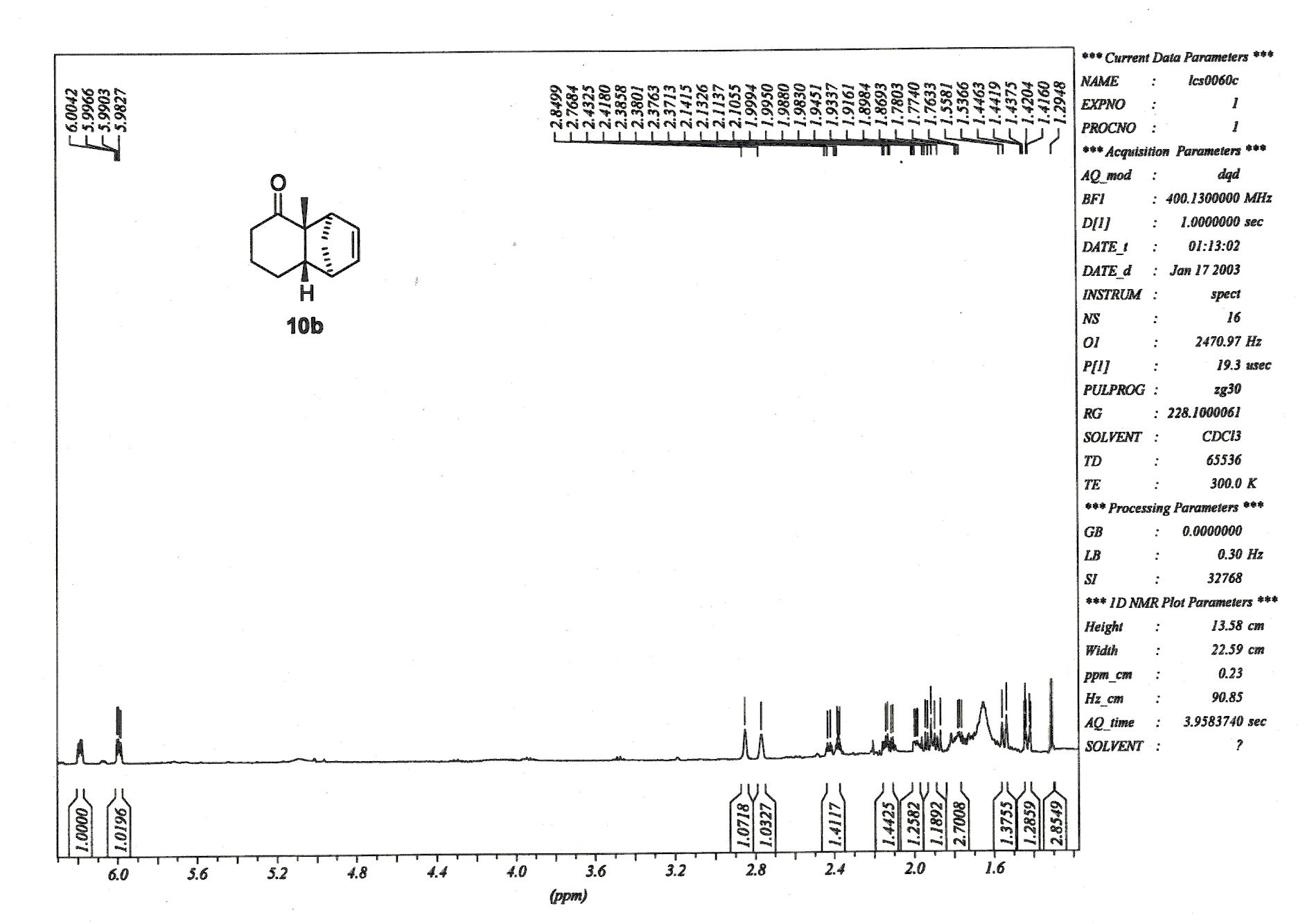

Supplement: File 2 — 1H and 13C NMR spectra of compounds 3, 4, 8ab, 9ab, 10ab, and 11a. (S2-S19). [file Beilstein_J_Org_Chem-01-14-s002.doc]
